# Future Prospective of Radiopharmaceuticals from Natural Compounds Using Iodine Radioisotopes as Theranostic Agents

**DOI:** 10.3390/molecules27228009

**Published:** 2022-11-18

**Authors:** Wiwit Nurhidayah, Luthfi Utami Setyawati, Isti Daruwati, Amirah Mohd Gazzali, Toto Subroto, Muchtaridi Muchtaridi

**Affiliations:** 1 Department of Chemistry, Faculty of Mathematics and Natural Sciences, Padjadjaran University, Sumedang 45363, Indonesia; 2 Department of Pharmaceutical Analysis and Medicinal Chemistry, Faculty of Pharmacy, Padjadjaran University, Sumedang 45363, Indonesia; 3 Research Collaboration Centre for Theranostic Radiopharmaceuticals, National Research and Innovation Agency (BRIN), Sumedang 45363, Indonesia; 4 Research Center for Radioisotope, Radiopharmaceutical, and Biodosimetry Technology, Research Organization for Nuclear Energy, National Research and Innovation Agency (BRIN), Serpong 15310, Indonesia; 5 School of Pharmaceutical Sciences, Universiti Sains Malaysia, USM, Penang 11800, Malaysia

**Keywords:** iodine radioisotopes, natural compounds, radiopharmaceuticals

## Abstract

Natural compounds provide precursors with various pharmacological activities and play an important role in discovering new chemical entities, including radiopharmaceuticals. In the development of new radiopharmaceuticals, iodine radioisotopes are widely used and interact with complex compounds including natural products. However, the development of radiopharmaceuticals from natural compounds with iodine radioisotopes has not been widely explored. This review summarizes the development of radiopharmaceuticals from natural compounds using iodine radioisotopes in the last 10 years, as well as discusses the challenges and strategies to improve future discovery of radiopharmaceuticals from natural resources. Literature research was conducted via PubMed, from which 32 research articles related to the development of natural compounds labeled with iodine radioisotopes were reported. From the literature, the challenges in developing radiopharmaceuticals from natural compounds were the purity and biodistribution. Despite the challenges, the development of radiopharmaceuticals from natural compounds is a golden opportunity for nuclear medicine advancement.

## 1. Introduction

Radiopharmaceuticals are drugs (pharmaceutical agents) that labeled with radioactive. They could be applied as theranostic agents. Radiopharmaceuticals are required to be target-specific, safe, and effective [1,2]. A radionuclide used for diagnostic purposes usually emits gamma rays, for example, technetium-99m, zirconium-89, indium-111, fluorine-18, xenon-133, iodine-123, and iodine-125. A radionuclide for therapeutic purposes emits alpha or beta rays, such as yttrium-90, iodine-131, samarium-153, lutetium-177, and astatine-211 [3,4]. In addition, the selection of radionuclides also considers half-life, energy, toxicity, and availability in nature. For example, ^111^In has the ideal SPECT imaging properties but has high cell DNA toxicity. Another example is zirconium-89, whose low pharmacokinetic properties are a perfect radionuclide for antibody labeling. However, the disadvantage is it can provide increased energy and penetrating photons during high abundance production [3].

A pharmaceutical agent can deliver a radiopharmaceutical to a target due to its specific and selective affinity for the target enzyme, protein, or receptor. The consideration in selecting a pharmaceutical agent is the ability to maintain its target specificity and selectivity after radiolabeling [5], and some of the common examples include small molecules such as NaI, peptides, and proteins. However, they still have various stability, specificity, and selectivity problems. These problems prompt the research for new pharmaceutical agents and among the class of compounds with the potential to be developed as natural-based compounds. Natural compounds are known as precursors with various pharmacological effects such as antioxidants, antibacterial, and anticancer activities. The affinity of natural compounds for disease targeting is also favorable in their development as pharmaceutical agents. To achieve this aim, suitable synthesis reactions that will allow stable binding of radionuclides to the natural compounds chosen are needed [6].

**Figure 1:**
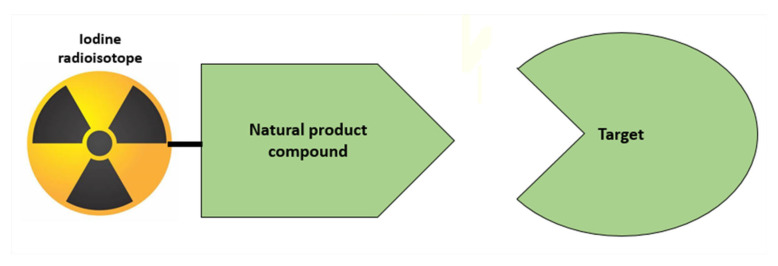
Basic scheme of natural-based radiopharmaceuticals with iodine radioisotope.

One of the radionuclides that are predicted to bind well with natural compounds is iodine isotopes [7]. Iodine has several isotopes, including iodine-123, iodine-124, iodine-125, and iodine-131 (Table 1). The labeling of natural compounds with iodine isotopes in the discovery of radiopharmaceuticals for diagnostics and therapeutics of various diseases is very promising. Natural compounds that act as pharmaceutical agents will deliver iodine isotopes to the target. The radiation emitted by the iodine isotopes will then function as either a diagnostic or a therapeutic agent (Figure 1). 

Although very promising, the development of radiopharmaceuticals from natural compounds using iodine isotopes is limited. More information needs to be gathered in order to understand the limitations and challenges of this approach, and to drive effective strategies for the research and development process. This review collects data of radiopharmaceuticals from natural compounds using iodine radioisotopes that were reported in the last ten years. The stages and the challenges were reviewed, and potential strategies were discussed to escalate the development of the radiopharmaceuticals. The information gained in this review will help researchers to advance the research and development of radiopharmaceuticals and nuclear medicine practice in the future.

## 2. Differences between Radiopharmaceuticals from Natural Compounds with Other Radiopharmaceuticals

Radiopharmaceuticals from natural compounds are radiopharmaceuticals that use natural compounds as the ligand. The ligand will then selectively interact with target tissues; thus, it has the ability to selectively deliver radionuclides. This interaction can occur pharmacologically, immunologically, or metabolically, and they may be reversible. After the interaction and the binding of the ligand with its target, the bonded radiopharmaceutical can be internalized and stored in the target cells. It is hence very crucial for the ligand to effectively, at a low concentration, prevent any pharmacological activity or side effects on the target [5].

In comparison with radiopharmaceuticals from natural compounds, conventional radiopharmaceuticals usually utilize small molecules, peptides, and proteins as ligands. Small molecules such as amino acids, fatty acids, nucleotides, and small inorganic molecules enable the targeting of intracellular regions because small molecules can penetrate semipermeable membranes easily. The examples of radiopharmaceuticals that use small molecules are [^123^I]NaI, [^123^I]ioflupane, and [123]] iobenguane for neuroblastoma tumors [5]. [^123^I]NaI is a substrate for sodium iodide symporter for thyroid imaging. The presence of a parathyroid adenoma is characterized by areas of cellular tissue which do not exhibit trapping of [^123^I]NaI [16]. [^123^I] ioflupane provides sensitive results in the diagnosis of Parkinson’s disease even in its early stages, based on the pattern of [^123^I]ioflupane uptake on SPECT images, which can be interpreted as normal activity if it shows no dopaminergic deficit [17]. [^123^I] iobenguana was applied to neuroblastoma tumors by targeting the norepinephrine transporter (NET) [18].

Radiopharmaceuticals that use peptides or proteins usually target specific receptors of tumor or cancer cells. Peptide cells can diffuse rapidly into target tissues and show longer accumulation in tumor cells. However, the disadvantage of using peptides or proteins as ligands is the potential for radio nephrotoxicity due to the high accumulation of peptides in the kidney. One example of a protein as a ligand is the Designed ankyrin repeat proteins (DARPins) labeled with iodine-124, iodine-125 and iodine-131 which is aimed to evaluate human epidermal growth factor receptor 2 (HER2) expression levels in breast and gastroesophageal cancer [19].

**Figure 2:**
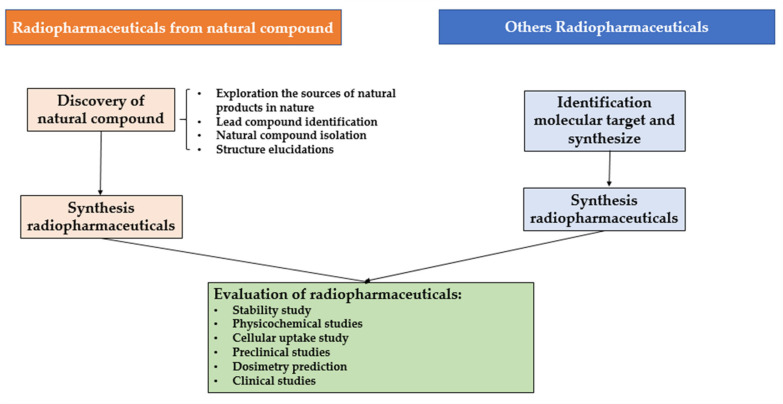
Development of radiopharmaceuticals from natural compounds in comparison to the conventional radiopharmaceuticals.

The difference between radiopharmaceuticals from natural compounds and other radiopharmaceuticals is also found in the stages of development of these drugs as shown in Figure 2. In general, the stages of development of other radiopharmaceuticals consist of identifying the molecular targets and synthesizing pharmaceutical compounds to be used as ligands (small molecules or peptides). Suitable radiopharmaceutical synthesis reaction will then be selected, and the evaluation of the synthesized radiopharmaceutical will be carried out. Meanwhile, radiopharmaceuticals from natural compounds tend to have a longer development stage. The initial stage of the development of radiopharmaceuticals from natural compounds is the discovery of natural compounds themselves. Research usually starts from exploring the sources of natural products in nature. After that, the lead compound will be identified, and the natural compounds will be isolated and identified by their structure elucidation. Subsequently, the molecular targets and pharmacological activities will be identified [6,20]. The next step is the selection of an appropriate radiopharmaceutical synthesis reaction based on the structure and the targets of the natural compounds. Some natural compounds also require structure modifications to get the best radiopharmaceutical synthesis results. Then, just like other radiopharmaceuticals, the radiopharmaceuticals from natural compound will also be characterized and evaluated based on the following criteria; the stability, physicochemical characteristics, cellular uptake, preclinical studies, dosimetry prediction, and clinical studies [6].

## 3. Available Literature on from Natural Compounds with Iodine Radioisotopes in Last 10-Year Period

**Figure 3:**
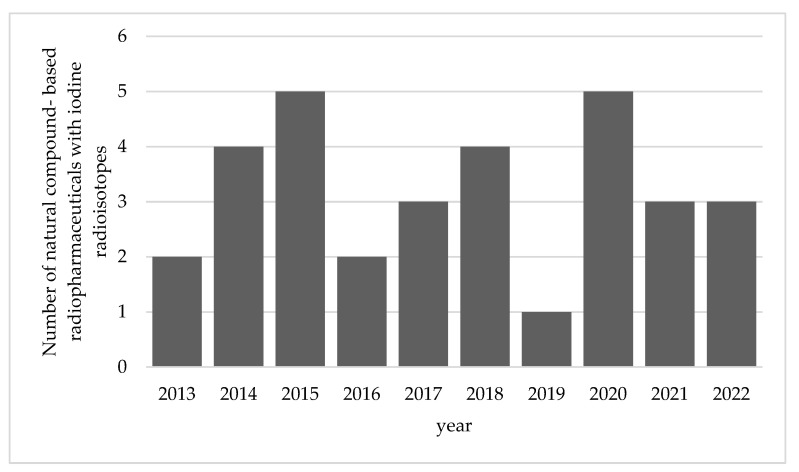
Research of radiopharmaceuticals from natural compounds in last 10 years.

This review summarizes the reported studies on radiopharmaceuticals from natural compounds with iodine radioisotopes in the last ten years, between 2013 and 2022. The data obtained from various research are presented in Figure 3.

From 2013–2022, 32 analyses on radiopharmaceuticals from natural compounds with iodine radioisotopes were conducted. The natural compounds are mostly isolated from plants, and their pharmacological effects are identified. These natural compounds are labeled with iodine radioisotopes to develop several therapeutic or diagnostic agents for various diseases, including nineteen for tumor or cancer, one for urinary tract dysfunction, one for Alzheimer’s disease, seven for necrotic myocardium, one for neuroblastoma, one for ischemic stroke, one for determination of natural compound toxicity, and another one was just labelled as radiotracer unspecified. As described in Figure 2, the discovery and development of radiopharmaceuticals have a long process. After conducting radiolabeling reaction or radiopharmaceutical synthesis, several evaluations need to be passed, including stability, physicochemical, cellular uptake, preclinical, dosimetry, and clinical studies. From the recent research based on the collected data from the 32 studies, 1 was in the synthesis stage, 1 was in the physicochemical study stage, 7 were in the cellular uptake study stage, 21 were in the preclinical study stage, 2 were in the dosimetry prediction stage and none of them reached the clinical study stage. Detailed information is listed in Table 2.

## 4. Synthesis of Radiopharmaceuticals from Natural Compounds with Iodine Radioisotopes

In the development of radiopharmaceuticals, researchers commonly favor two kinds of synthesis reactions: (1) synthesis with nonradioactive iodine (iodine-127), and (2) radiosynthesis with iodine radioisotope. The purpose of nonradioactive synthesis is to predict the structure of the objective compound by elucidating the structure using MS (Mass Spectroscopy) and NMR (Nuclear Magnetic Resonance). Radiosynthesis was to be tested for radiochemical purity. They are usually carried out through two reaction mechanisms, namely (1) electrophilic substitution and (2) nucleophilic substitution [7,93].

### 4.1. Electrophilic Substitutions

Electrophilic substitution reactions occur when iodine substitutes hydrogen on electron-rich aromatic rings such as phenol and group-substituted benzene rings. In general, iodine is available in the form of NaI solution therefore it needs to be converted into an electropositive form before reacting with pharmaceutical compounds using oxidizing agents, iodo–deprotonation, and iodo–demetallation. The oxidizing agent converts iodine to its electropositive form by oxidizing it thus its oxidation number increases. There are two types of oxidizing agent: (1) oxidizing agents containing halogens, and (2) oxidizing agents without halogens. Oxidizing agents with halogens include chloramine-T, iodine, and *N*-chlorosuccinimide, meanwhileoxidizing agents without halogens include tert-butyl hydroperoxide, peracetic acid and hydrogen peroxide [94]. Iododeprotonation usually occurs in aromatic compounds that have an electron-rich ring activated by OH, NH_2_, or OMe [95]. Iododemetallization is reaction using organometallic precursors such as trialkylstannyl, trialkylsilyl, or boronic acid derivatives [93].

### 4.2. Nucleophilic Substitution

Nucleophilic substitution reactions consist of several methods, including halogen exchange, isotope exchange, radioiodo-dediazonisation, and copper-assisted halogen exchange. The halogen exchange method occurs when radioactive iodine substitutes a halogen (bromine or chlorine) in the pharmaceutical compound [93]. This reaction requires extreme conditions. Zmuda et al. (2015) synthesized a tracer for Poly (ADP-ribose) Polymerase-1 (PARP-1) with solid state halogen exchange radioiodination method using bromination. The reaction took place under extreme conditions whereby the reaction temperature was 210 °C with an incubation time of 0.5 h. The authors reported the radiochemical yield obtained was 36.5 ± 7.2% [93,96].

The isotope exchange reaction was carried out by substituting the iodine present in the ligand with iodine radioactive. This reaction usually occurs under reflux with solvents. The solvents used are acetone, dichloromethane, acetonitrile, water, ethanol, or methyl ethyl ketone. Sadeghzadeh et al. synthesized 4-benzyl-1-(3-[^125^I]iodobenzylsulfonyl)piperidine and 4-(3-[^125^I]iodobenzyl)-1-(benzylsulfonyl)piperazine using this reaction. It used a wet method using different organic solvents, such as propylene glycol at elevated temperatures (100–200 °C) where the results showed that the purity obtained was 70% [93,97]. Radioiodo–dediazonisation is a radioiodination method of compounds with a diazonium group. The reaction was conducted by substituting diazonium with radioiodine. It is usually carried out at low temperatures with the help of sodium nitrate. The reaction proceeds by the SN1 mechanism [93]. Copper-assisted exchange is a nucleophilic substitution reaction that uses copper as a catalyst. The reaction can occur via isotopic or halogen exchange. M. Hagimori et al. synthesized matrix metalloproteinase-12 (MMP-12) using a copper-assisted exchange. The reaction was conducted at 140 °C for 60 min, with high purity product [93,98].

### 4.3. Synthesis of Radiopharmaceuticals from Natural Compound with Iodine Radioisotopes in the Last 10 Years

The synthesis of radiopharmaceuticals from natural compounds is a challenging process. Natural compounds are expected to be labeled as stable and produce high radiochemical purity. The natural compounds to be labeled are mostly isolated from plants. Prior to labeling, they are usually characterized by LC/MS, HPLC, or NMR. Based on the literature study, several research articles reported the characterization method of natural compounds, but several articles have not reported it. Out of the 32 radiopharmaceuticals of natural compounds using iodine radioisotope, 17 reported the natural compound characterization method, while 15 have not reported it. From the data collected in the last 10 years, all reported compounds were synthesized through electrophilic substitution reactions. This is because natural compounds would usually have an electron-rich aromatic ring. Of the 32 labeled compounds, 31 were synthesized in the presence of oxidizing agents, and 1 through iodo–destannylation. As oxidizing agents, 20 compounds used iodogen, 9 with chloramine-T, and 2 compounds with peracetic acids. Detailed information is listed in Table 3.

**Figure 4:**
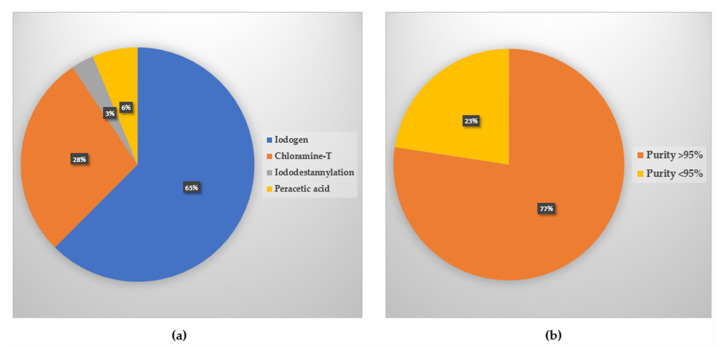
(**a**) Synthesis method and type of oxidizing agents used; (**b**) Purity of the 32 candidates of natural compound-based radiopharmaceuticals.

The synthesis method and type of oxidizing agent used in the synthesis, and the purity of the 32 radiopharmaceutical candidates are described in Figure 4. The results of radiosynthesis showed that 24 compounds (77%) had radiochemical purity above 95% while 8 (23%) had a purity lower than 95%.

## 5. Evaluations of Radiopharmaceuticals from Natural Compounds with Iodine Radioisotopes

As illustrated in Figure 2, the evaluation of radiopharmaceuticals includes stability tests, physicochemical analysis, cellular uptake study, preclinical study, dosimetry prediction, and clinical study. All radiopharmaceuticals, including those derived from natural compounds, will need to go through these evaluations before approval can be granted for human use. Table 4 presents the evaluation of the 32 radioiodinated natural compound as collected and analyzed for this review. From the data, 22 of them reported stability tests, 8 of them reported physicochemical analysis, 10 of them reported cellular uptake study, 23 of them reported preclinical study, 2 of them reported dosimetry prediction and non-reported clinical study.

The stability of radiopharmaceuticals is affected by several factors such as pH, light, and temperature. It needs to be stored under various storage conditions [98]. From 22 radioiodinated natural compounds that reported stability tests, the stability was classified into two groups: stability ≥ 24 h and <24 h, with a total of 14 and 8 compounds, respectively.

The physicochemical analysis consists of lipophilicity and protein binding characterizations. Lipophilicity, quantified as Log D or Log P, is a crucial parameter in estimating radiopharmaceuticals absorption, distribution, metabolism, and excretion (ADME) [99,100]. Besides lipophilicity, protein binding affects the biodistribution and clearance of radiopharmaceuticals. It has a positive correlation with lipophilicity [101].

**Figure 5:**
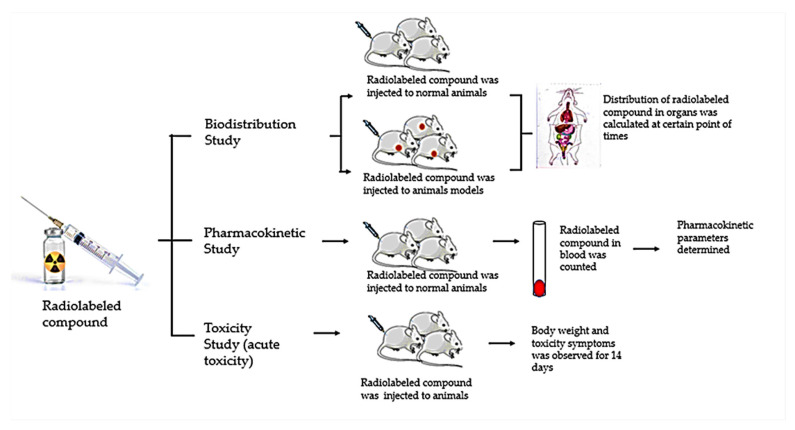
Preclinical studies of radiopharmaceuticals.

Cellular uptake study aims to determine the specificity of a radiopharmaceutical towards its target by using cells or tissues that expresses the target [4,102]. The tested radiopharmaceutical will be incubated with cultured cells and cell uptake will be calculated as the percentage of radioactivity in cells compared to total radioactivity [103]. The selection of cell lines used depends on the specific target of the radiopharmaceutical on the receptor or on certain physiological conditions. Some of the cell lines that are often used include: Hutu80 (human gastrointestinal tumor cell lines), Caco-2 (human colon adenocarcinoma cells), MCF7 (human breast adenocarcinoma cells), PC3 (human prostate carcinoma cells), Keratinocyte (Human normal epidermal keratinocyte cells), BJ (Human normal foreskin fibroblast cells), TT, FTC-133, and DRO (human thyroid cell lines), SK-N-AS and SH-SY5Y (human neuroblastoma cell lines), MDA-MB-231 (the triple-negative breast cancer cell lines), and SKOV3 (human ovarian cancer cell lines). After cellular uptake, preclinical study will be conducted using experimental animals. In general, it consists of biodistribution, pharmacokinetics, and toxicity studies. A biodistribution study will allow the determination of radiopharmaceutical uptake in the animal organs, which will be calculated as %ID/g [104]. Based on the reported biodistribution study data, several compounds ([^131^I]hydroxytyrosol, [^123^I]hesperetin, [^125^I]rutin, [^131^I]khellin, and [^125^I]zearalenone) have a high accumulation pattern in certain organs, especially the thyroid, intestine and stomach. A pharmacokinetic study is needed to determine the pharmacokinetic parameters such as elimination rate constant (K_e_), the volume of distribution (Vd), area under the curve [105], and clearance and time to maximum concentration (T_max_) [106]. Radiopharmaceuticals are expected to have rapid blood clearance and short t_1/2_ elimination so that they can be excreted rapidly from the blood. Based on data collected, [^131^I]sennidin A, [^131^I]protohypericin, [^131^I]sennoside B, [^131^I]rhein, [^131^I]vitexin, [^131^I]napthazarin, and [^131^I]shikonin reported pharmacokinetic study with t_1/2_ elimination value of 11.75, 14.9, 8.6, 8.2, 5.3, 4.73, and 0.675 h, respectively. Toxicity study aims to evaluate the safety of radiopharmaceuticals. Koziorowski et al. (2016) stated that in radiopharmaceuticals, acute toxicity tests were carried out to predict the effect of overdose whereas subacute, chronic, teratogenic, mutagenic and carcinogenic toxicity were not required for radiopharmaceuticals [107]. The schematic diagram of the preclinical study is depicted in Figure 5.

Dosimetry prediction is a procedure in the determination of the absorbed dose as the amount of energy absorbed per unit mass in all irradiated tissues or organs of interest. The aim was to determine the reference levels of irradiation for every new radiopharmaceutical or estimate the absorbed dose for routinely used radiopharmaceuticals [108]. The final stage of radiopharmaceutical development is clinical study. This stage is carried out based on the regulations set in each region or country because, in general, each region or country would have different regulations regarding the rules of radiopharmaceutical-based clinical trials [5].

## 6. Challenge and Strategies

The development stages of radiopharmaceuticals from natural compounds with iodine radioisotope are rather long and challenging. The challenges reported in previous studies were mostly related to radiochemical purity and biodistribution, as shown in Table 5. However, some radiolabeled compounds such as [^131^I]genistein have not been tested in vitro and in vivo, so further development is needed.

### 6.1. Problem Related to Radiochemical Purity and the Strategies

Radiochemical purity is a crucial quality control factor in radiopharmaceutical development. The radiochemical impurities of iodine are free I^−^ and I_2_ which affect the safety and accuracy of radiopharmaceuticals. The first strategy to obtain the maximum radiochemical purity is to select a suitable radioiodination method based on the steric characteristics of the natural compound as a substrate to be labeled. In addition, critical point optimization in the radioiodination method is important because it can minimize the formation of impurities. The first strategy is to optimize the critical point of radioiodination. [7,94]. Substrate characteristics and critical reaction points should be considered in selecting the radioiodination reaction method, as summarized in Table 6.

Selection of the suitable radioiodination method and optimization of the critical point in radioiodination could lead to high radiochemical purity. However, if the radiochemical purity is still lower than the required purity (>95%), another strategy that can be conducted is purification to separate impurities from the radiopharmaceuticals. The selection of the purification method depends on the molecular weight, lipophilicity, and molecular charge of the radiopharmaceuticals. Purification methods that can be applied include HPLC (High-Performance Liquid Chromatography), SPE (Solid Phase Extraction), SEC (Size-Exclusion Chromatography), and IEC (Ion-Exchange Chromatography.

One of the most widely used purification methods is the HPLC. The separation of compounds occurs due to differences in solute interactions and the column that lead to different elution rates for each component. As a result, it will provide high purity resolution. The parameters to consider in HPLC purification are polarity, flow rate, pH, the lipophilicity of the mobile phase, sample matrix, type of stationary phase, and temperature. SPE is widely chosen because it is simple, fast, and able to separate dissolved or suspended compounds from other compounds in the mixture based on their physical and chemical properties. Kim et al. (2019) conducted purification of [^131^I]metaiodobenzylguanidine using solid phase extractionand obtained a higher amount of product and lower exposure of operator to radiation [122]. SPE is commonly used in the separation of macromolecules that consist of substances with different molar masses. The SEC chromatography column uses porous polymeric beads. The pore size determines the dimensions of the compounds to be separated. Molecules with smaller size than the pores can enter the pores and retain, while the molecule with larger size than the pores will pass through the spaces between the packing material. In this way, the molecules with the highest molecular weight will be obtained in the first fraction. Lemps et al. performed purification of [^123^I]bevacizumab using the SEC method and obtained a radiochemical purity of 99.5% after purification [123]. IEC is a separation method for ions and polar molecules [124]. IEC consists of anion and cation exchange. Cation-exchange chromatography uses a negatively charged stationary phase that can separate cations from other ions. By contrast, anion-exchange chromatography uses a positively charged stationary phase that can separate anions from the other ions. Before conducting an IEC, the stationary phase should be achieved through electroneutrality. Visser et al. performed a purification of [^131^I]c-MOv18 which was a radiopharmaceutical candidate for therapy for ovarian cancer. The impurities were removed by purification using Dowex AG1-X8 (BioRad, Utrecht, The Netherlands) anion-exchange resin in PBS [125,126].

### 6.2. Problem Related to Biodistribution and the Strategies

Ideally, radiopharmaceuticals are required to have high specificity, rapid accumulation in target organs, and a high target-to-nontarget ratio [127]. Based on previous studies, some labeled compounds showed high biodistribution in other organs compared to the targeted organs. Altered biodistribution in vivo affected the accuracy of imaging and radiopharmaceutical therapy. This problem occurred due to the presence of other compounds, such as impurities (free I^−^) or residues that could be uptaken in organs and detected as radiopharmaceuticals. Spetz et al. conducted a study to determine the biodistribution of free ^125^I^−^ and ^131^I^−^ in rats and reported the highest biodistribution in the thyroid gland and stomach. In primary conditions, iodine was localized in the thyroid. In addition, the iodine uptake in the stomach occurred due to the expression of an iodine transport medium named Na+/I− sodium iodide symporter in the stomach [128].

The formation of impurity free iodide indicates low in vivo stability of C-I in the radiopharmaceuticals due to the deiodination reaction. The accumulation of free iodide as impurity in the thyroid, stomach, and intestine reduces the target-to-background ratio of the diagnostic agent so that the diagnostic results are biased. In therapeutic applications, it increases the accumulation of radioactivity in the non-target organs, which could lead to adverse reactions in these healthy organs. This in vivo deiodination reaction is caused by several enzymes including deiodinase enzymes, cytochromes P450 (CYP450) enzymes, and nonspecific nucleophilic enzymes. Deiodinase enzymes promote deiodination reactions in iodinated aromatic rings such as ortho–iodo–phenols. CYP450 enzymes promote deiodination via xenobiotics oxidation reactions, whereas nonspecific nucleophilic enzymes promote deiodination at electrophilic carbon atoms [129].

The first strategy to decrease free iodine accumulation in non-target organs is to design radiopharmaceuticals resistant to deiodination reactions with modification structural. In general, compounds with an arene group are stable to deiodination. In the iodination of the arene group, metaiodoarene is more resistant to deionization than orthoidoarene and paraiodoarene. In addition, iodination at sp^2^ carbon atoms is usually more stable to deiodination reactions than iodination at sp and sp^3^ carbon atoms. Radioiodination of the vinyl group is also stable against in vivo deiodination reactions. However, the phenol and aniline groups have poor in vivo stability. Their stability can be improved by adding electron-donating substituents such as OCH_3_ to the aromatic ring. On the other hand, the addition of electron-withdrawing groups can decrease in vivo stability [129]. The resistance of some radioiodinated groups against in vivo deiodination is listed in Table 7.

**Figure 6:**
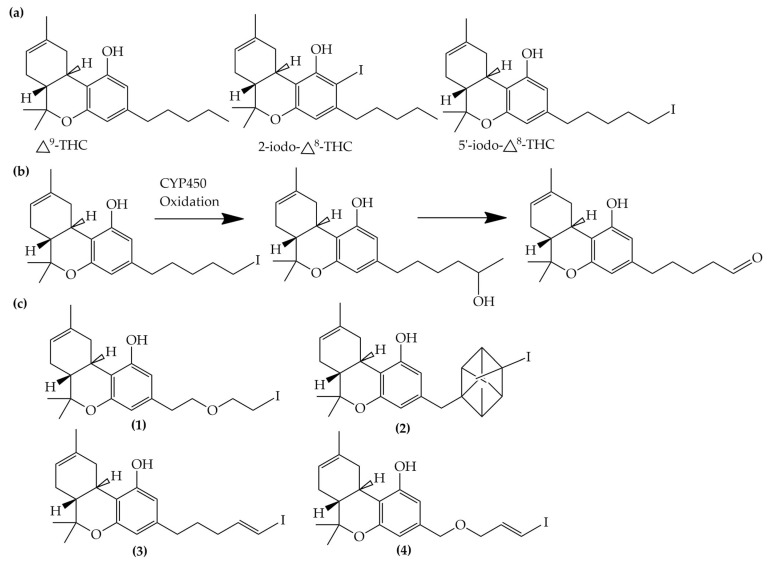
(**a**) Structure of ∆^9^-THC, 2-iodo-∆^8^-THC and 5′-iodo-∆^8^-THC; (**b**) The mechanism of deiodination reaction of 5′-iodo-∆^8^-THC; (**c**) Recommendation structure of iodinated THC which is stable against deiodination reaction [130].

Compton et al. (1993) conducted the radioiodination of ∆^9^-Tetrahydrocannabiol (∆^9^-THC) to produce 2-iodo-∆^8^-THC and 5′-iodo-∆^8^-THC. ∆^9^-THC is a natural compound isolated from *Cannabis Sativa*. The target of radioiodinated ∆^9^-THC is the imaging cannabinoid system. The structure of ∆^9^-THC, 2-iodo-∆^8^-THC, and 5′-iodo-∆^8^-THC are shown in Figure 6a. The in vivo study of 5′-iodo-∆^8^-THC showed a poor in vivo profile due to the deiodination reaction (shown in Figure 6b). The position of iodine on a terminal sp3 carbon atom of linear pentyl moiety allows 5′-iodo-∆^8^-THC to be susceptible to in vivo deiodination caused by CYP450. Cavina et al. (2016) provide solutions for structural modifications that are expected to increase the stability against deiodination, including the position of iodine on the iodo-ethoxy group, iodine on the cubane position, iodine on carbon sp2 at the vinyl terminal, or iodine on C sp2 in the terminal allyl moiety [129]. The structural modification is shown in Figure 6c. Based on this case, structural modification of natural compounds provides a promising strategy to increase the stability of radioiodinated natural compounds against in vivo deiodination.

The second strategy to increase the in vivo stability of natural compound-based radiopharmaceutical candidates is to label them with a linker. This linker will form a stable chemical bond between the iodine radioisotope and the natural compounds [5], and it must have a good in vivo stability. Kim et al. (2016) conducted radioiodination of cetuximab with the linker (*N*-(4-isothiocyanatobenzyl)-2-(3-(tributylstannyl)phenyl) acetamide (IBPA). It was reported that [^125^I]IBPA-cetuximab had a more stable binding and higher internalization in mice bearing LS174T tumor xenografts compared to [^125^I]cetuximab [130].

**Figure 7:**
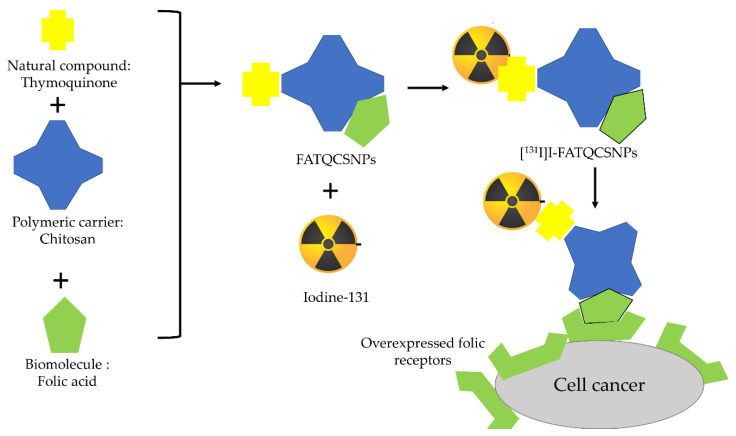
A schematic diagram of [^131^I]FATQSCNPs.

Another strategy that can be applied is to increase the target specificity by using nanoparticles. Nanoparticles play a role in increasing the penetration of compounds across biological membranes so that they can effectively deliver therapeutic agents and reduce the side effects of conventional delivery techniques. Nanoparticles can increase the delivery specificity of radiopharmaceuticals towards its target by conjugating the targeting molecule (ligand) on the nanoparticles’ surface [131]. This technique was carried out by Ince et al. (2016) who produced the [^131^I]FATQCSNPs (folic acid-chitosan nanoparticles loaded with thymoquinone) described earlier, with ovarian cancer cells as the target. [^131^I]FATQCSNPs incorporated thymoquinone isolated from *Nigella sativa* in a folic acid-chitosan nanoparticles [74]. Folic acid is a small molecule that is useful as a ligand that helps in the internalization of pharmaceuticals into cancer cells [75]. Folic acid was used due to ovarian cancer cells that were marked with overexpression of folic acid. The encapsulation of thymoquinone within folic acid-chitosan nanoparticles has improved the delivery of thymoquinone, especially with the presence of folic acid that helps to increase the specificity of delivery and increase cellular uptake. Both [^131^I]thymoquinone and [^131^I]FATQSCNPs were developed for diagnostic and therapy of cancer. The radioiodinated complex showed a higher uptake in SKOV3 cells as compared to [^131^I]thymoquinone [76]. A schematic diagram of [^131^I]FATQSCNPs is shown in Figure 7.

## 7. Methods

This review was conducted based on the results of the collection and analysis of articles obtained from the PubMed database with the following keywords: “radioiodination of natural compound”; “radiopharmaceutical natural compound”; “radioiodination of flavonoid”; “radioiodination of alkaloid”; “radioiodination reaction mechanism”; “design AND challenge new radiopharmaceutical”; “radiopharmaceutical AND natural product AND iodine”.

The inclusion criteria of the main article were articles that discuss radioiodination of natural compounds using the English language and published within the range years of 2013-2022. The inclusion criteria of the supporting articles were articles that discuss radiopharmaceuticals in general, iodine isotopes, and the pharmacological effects of natural compounds. Exclusion criteria were articles that were published more than 10 years ago for main articles, 20 years ago for supporting articles, and non-relevant articles to the topic discussed in this review.

**Figure 8:**
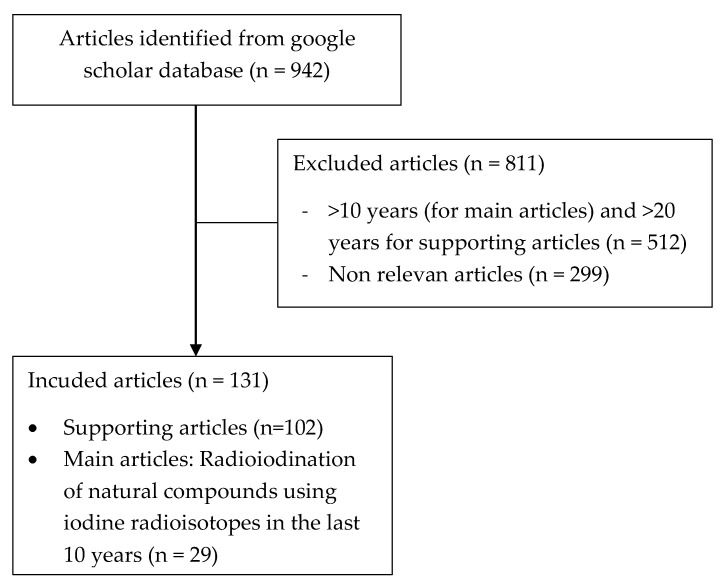
The literature search flow.

Based on the search conducted using the aforementioned keywords, 942 journals were obtained and 512 articles were discarded as they were published more than 10 years ago (for main articles) and 20 years ago (for supporting articles), while 299 articles were non-relevant articles to the topic discussed in this review. This step has reduced the number of articles to 131 consisting of 102 supporting articles and 29 articles discussing the radioiodination of natural compounds with iodine radioisotope. The literature search flow is shown in Figure 8.

## 8. Future, Prospect, and Conclusions

The development of radiopharmaceuticals from natural compounds with iodine radioisotope is a long process with several challenges. Thirty-two radioiodinated natural compounds were collected from a literature study of the last 10 years. To determine the challenges that radiopharmaceutical researchers found in natural compounds, we reviewed 32 compounds from their synthesis to their evaluation results. These challenges are clasified into two groups: (1) challenges related to chemical purity, and (2) challenges related to biodistribution. We discussed strategies that could be applied to resolve these challenges.

Based on the data, 8 of the 32 radioiodinated natural compounds collected had radiochemical purity problems. The first strategy offered is to optimize the critical point in the synthesis reaction to obtain the optimum synthesis conditions. The second strategy is the purification of synthetic products in several ways, including High-Performance Liquid Chromatography (HPLC), SPE (Solid Phase Extraction), (SPE), SEC (Size-Exclusion Chromatography), and IEC (Ion-Exchange Chromatography). The purification method can separate the product from impurities.

Based on the evaluation results, five radioiodinated natural compounds have problems with their biodistribution. Unspecified accumulation is characterized by high accumulation in the stomach, intestines, and thyroid. This unspecific accumulation shows poor in vivo stability due to the deiodination reaction. Several strategies to solve this problem include designing radiopharmaceuticals resistant to in vivo deiodination by structural modification, radioiodination with linkers, and application of nanoparticles.

Despite the challenges, the development of radiopharmaceuticals from natural compounds using iodine radioisotope offers a bright future in the development of radiopharmaceuticals. This review provides information that researchers undertaking further research can consider. The strategies offered in this review are expected to encourage improvement in research related to natural product-based radiopharmaceuticals with iodine radioisotopes as theranostic agents for various diseases.

## Figures and Tables

**Table 1:** Radioisotopes of iodine.

Radioisotope	Half Life	Emission Type	Application	Refs.
iodine-123	13.2 h	Gamma, EC ^1^/auger	SPECT ^3^ diagnostic	[8,9,10]
iodine-124	4.8 days	Positron	PET ^2^ diagnostic	[11,12,13]
iodine-125	60 days	Gamma, EC ^1^/Auger	Preclinical study, Radiotherapy SPECT ^3^ diagnostic	[14,15]
iodine-131	8.04 days	Gamma, beta	Radiotherapy, SPECT^3^ and PET ^2^ diagnostic	[8,9,10]

^1^ EC: Electron capture, ^2^ PET: Positron emission tomography, ^3^ SPECT: Single photon emission tomography.

**Table 2:** Recent report of radiopharmaceuticals from natural compound with iodine radioisotopes and their importance of development/ application.

Year	Natural Compounds	Sources	Pharmacological Activities	Radioisotope	Application	Recent Research Reported	Refs.
2013	Hydroxytyrosol	olive leaves extract	Anticancer (breast, colon, prostate, and thyroid cancer)	iodine-131	Cancer therapy	Preclinical study	[21,22,23,24,25,26]
	Khellin	*Ammi visnaga* fruits	Activity against kidney disease and vitiligo, anticancer	iodine-125	Urinary tract imaging	Preclinical study	[27,28]
2014	Hypericin	*Hypericum perforatum* L.	Antiviral, necrosis avidity and anticancer	iodine-131	Cancer therapy	Dosimetry prediction	[29,30]
	Hypericin	*Hypericum perforatum* L.	Antiviral, necrosis avidity and anticancer activity	iodine-123	Cancer therapy	Dosimetry prediction	[30,31]
	Lawsone	*Lawsonia inermis*	Anticancer, antioxidant, and antibacterial	iodine-131	Cancer theranostic	Preclinical study	[32,33,34]
	Homoisoflavonoids	*Hyacinthaceae* and *Caesalpinioideae*	Formation, extension, and destabilization of Aβ aggregates	iodine-125	diagnostic of b-amyloid plaques in Alzheimer’s disease	Preclinical study	[35]
2015	Gingko flavonoids (GFLAs)	Egb761 extract of *Gingko Biloba*	Anticancer	iodine-131	Cancer diagnostic	Cellular uptake	[36]
	Sinnidine A	*Cassia Senna* L.	Structure similar to hypericin so it is predicted to have necrosis affinity like hypericin	iodine-131	Myocardial infarction imaging	Preclinical study	[37]
	Protohypericin	*Hypericum perforatum*	Structure similar to hypericin so it is predicted to have necrosis affinity like hypericin	iodine-131	Cancer theranostic	Preclinical study	[38]
	Sennoside B	*Cassia senna* L.	Structure similar to hypericin so it is predicted to have necrosis affinity like hypericin	iodine-131	Necrosis-avid tracer	Preclinical study	[39]
	Hesperetin	*citrus* fruits	Anti-inflammatory, antioxidant, anticancer, antiviral, antiallergic, and neuroprotective	iodine-123	Radiotracer for some disease	Preclinical study	[40,41,42]
2016	Rutin	*citrus* leaves	Antitumor, cytotoxic, anti-inflammatory, antiestrogenic, antimicrobial, antiallergic, and antioxidant	iodine-125	Cancer diagnostic	Preclinical study	[43,44]
	Rhein	*Cassia fistula* L.	Necrotic myocardium	iodine-131	Myocardium necrosis imaging	Preclinical study	[45,46,47]
2017	Eugenol	*Syzygium aromaticum*	Anticancer (prostate, breast, colon, and cervical cancer)	iodine-131	Cancer therapy	Cellular uptake	[48,49,50,51]
	Quercetin	vegetables, fruits, leaves, and grains	Anticancer	iodine-131	Thyroid cancer therapy	Preclinical study	[52,53,54]
	Arbutin	fresh fruit of the *California buckeye*	A tyrosinase inhibitor and antitumor	iodine-131	Tumor diagnostic	Preclinical study	[55,56,57]
2018	Vitexin	*Passiflora caerulea* L.	Necrosis-avid activity	iodine-131	Myocardium necrosis imaging	Preclinical study	[58]
	Napthazarine	green walnut husks of *Juglans Mandshurica Maxim*	Necrosis-avid activity	iodine-131	Myocardium necrosis imaging	Preclinical study	[59,60]
	Plumbagin	*Plumbago zeylanica*	Necrosis-avid activity	iodine-131	Myocardium necrosis imaging	Preclinical study	[60,61]
	Juglone	leaves and nuts of various plants from the Juglandaceae family	Necrosis-avid activity	iodine-131	Myocardium necrosis imaging	Preclinical study	[60,62]
2019	Resveratrol	grapes, peanut, and *Polygonum cuspidatum* root	Anti-inflammatory, antiapoptotic, neuroprotective antitumor, and immunological regulatory	iodine-131	Neuroblastoma cells imaging	Cellular uptake	[63,64,65,66,67]
2020	Genistein	Soybeans	Anticancer (Breast cancer)	iodine-131	Breast cancer diagnostic	Synthesis	[68,69,70,71]
	6-Gingerol	ginger-roots extract	Anticancer (breast cancer)	iodine-131	Breast cancer diagnostic	Cellular uptake	[72,73,74]
	6-Shogaol	ginger-roots extract	Anticancer (breast cancer)	iodine-131	Breast cancer diagnostic	Cellular uptake	[72,73,74]
	Thymoquinone	*Nigella sativa*	Anticancer	iodine-131	Cancer theranostic	Cellular uptake	[75]
	FATQCSNPs (Folic acid-chitosan nanoparticles loaded with thymoquinone)	*Nigella sativa*	Anticancer	iodine-131	Cancer theranostic	Cellular uptake	[75,76,77]
2021	Rutin	Several fruits and vegetables	Anticancer	iodine-131	Cancer diagnostic	Physicochemical study	[78]
	Ferulic acid	Several fruits and vegetables	Anticancer, antidiabetic, and activity against several neurodegenerative and cardiovascular diseases	iodine-131	Cancer theranostic	Preclinical study	[79,80,81,82]
	Khellin	*Ammi visnaga* fruits	Anticancer	iodine-131	Cancer theranostic	Preclinical study	[83]
2022	Zaeralenone	cereal crops	Ability to bind competitively with estrogen receptors	iodine-125	to study the the effect of *Lactobacillus Plantarum* on biodistribution pattern of Zaeralenone	Preclinical study	[84,85,86]
	Riboflavin	meat, fish and fowl, eggs, dairy products, green vegetables, mushrooms, and almonds	Activity against nervous system diseases	iodine-131	Ischemic stroke diagnostic	Preclinical study	[87,88,89]
	Shikonin	*Lithospermum erythrorhizon*	Anticancer (lung cancer)	iodine-131	Lung cancer diagnostic	Preclinical study	[90,91,92]

**Table 3:** Characterization of natural compounds and synthesis of radiopharmaceuticals candidates from natural compounds using iodine radioisotope that were reported in the last 10 years (2013–2022).

Natural Compound	Characterization	Synthesis	Iodinated Natural Compound	Characterization	Refs.
Hydroxytyrosol 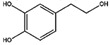	LC-MS (liquid chromatography-mass spectrometry) with positive mode [M+H] showed *m*/*z* 155.	iodogen	[^131^I]hydroxytyrosol 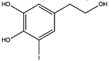	Structure was characterized by ^1^H NMR and ^13^C NMR Radiochemical Purity > 95% (by TLRC)	[26]
Khellin 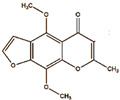	Not reported	chloramine-T	[^125^I]khellin 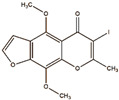	Radiochemical Purity < 95% (by TLRC)	[28]
Hypericin ^ 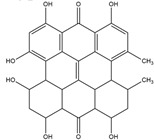 ^	HPLC-UV with retention time of 7.85 min	iodogen	[^131^I]hypericin 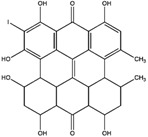	HPLC with retention time of 11.57 min Radiochemical Purity: >95% (by HPLC)	[29,30]
Hypericin ^ 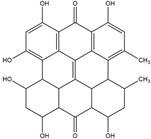 ^	HPLC-UV with retention time of 7.85 min	iodogen	[^123^I]hypericin 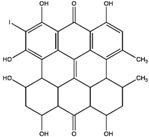	HPLC with retention time of 11.57 min Radiochemical Purity: >95% (by HPLC)	[30,31]
Lawsone 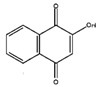	Structure was characterized by ^1^H NMR and ^13^C NMR	iodogen	[^131^I]lawsone 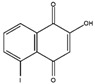	Structure was characterized by ^1^H NMR and ^13^C NMR Radiochemical Purity: <95% (by TLRC)	[33]
Homoisoflavonoid ^ 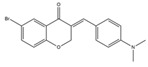 ^	Structure was characterized by ^1^H NMR and ^13^C NMR	iododestannylation	[^125^I]I-Homoisoflavonoid 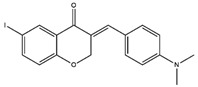	Structure was characterized by ^1^H NMR and ^13^C NMR -Radiochemical Purity: >95% (by HPLC)	[34]
GFLAS	Characterized by HPLC	iodogen	[^131^I]GFLAS Predicted structure have not reported	Radiochemical Purity: <95% (by TLRC)	[36]
Sennidin A ^ 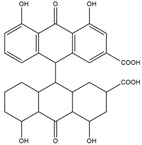 ^	HPLC-UV with retention time of 9.98 min	iodogen	[^131^I]sennidin A 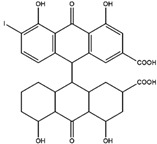	HPLC-UV with a retention time of 11.76 min Radiochemical Purity: <95%	[37]
Protohypericin ^ 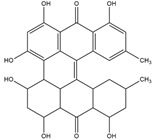 ^	HPLC-MS/MS [M,H]^-^ with *m*/*z* 505 Structure was characterized by ^1^H NMR and ^13^C NMR	iodogen	[^131^I]protohypericin 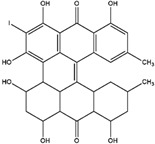	Radiochemical Purity: >95% (by HPLC)	[38]
Sennoside B ^ 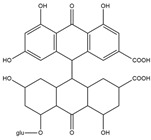 ^	HPLC with retention time of 7.09 min	iodogen	[^131^I]sennoside B 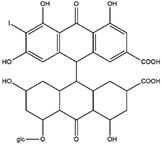	HPLC with retention time 9.55 min Radiochemical Purity: >95% (by HPLC)	[3]
Hesperetin ^ 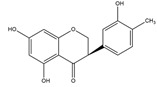 ^	LC/MS with [M,H]^+^ show *m*/*z* of 427 Structure was characterized by NMR	peracetic acid	[^123^I]hesperetin 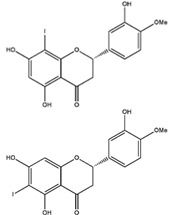	Structure characterized by NMR and COSY analysis Radiochemical Purity: >95% (by HPLC)	[42]
Rutin ^ 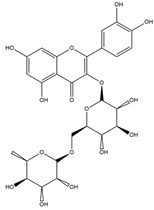 ^	Structure was characterized by NMR	chloramine-T	[^125^I]rutin 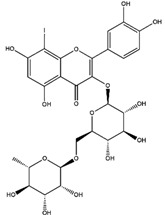	Structure was characterized by NMR LC MS [M+H]+ with *m*/*z* of 737 Radiochemical Purity: >95% (by HPLC)	[44]
Rhein ^ 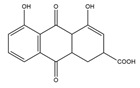 ^	Not reported	peracetic acid	[^131^I]rhein 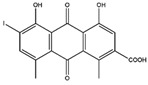	Structure was characterized by NMR LC MS [M-H]- with *m*/*z* of 408.9 Radiochemical Purity: >95% (by HPLC)	[47]
Eugenol ^ 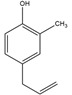 ^	LC MS [M+H]^+^ with *m*/*z* of 164.80 HPLC with retention time of 12.456 min	iodogen	[^131^I]eugenol 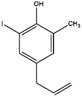	Structure was characterized by NMR Radiochemical Purity: >95% (by TLRC)	[51]
Quercetin ^ 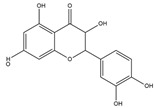 ^	Not reported	chloramine-T	[^131^I]quercetin 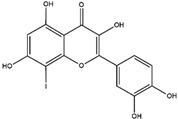	LC/MS characterization Radiochemical Purity: >95% (by HPLC)	[54]
Arbutin ^ 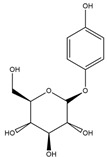 ^	HPLC with retention time of 1.6 min	chloramine-T	[^131^I]arbutin 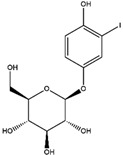	HPLC with retention time of 19,9 min Radiochemical Purity: >95%	[57]
Vitexin ^ 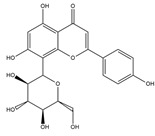 ^	Not reported	iodogen	[^131^I]vitexin 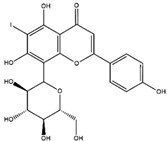	Stucture was characterized by NMR Radiochemical Purity: >95% (by HPLC)	[58]
Napthazarine ^ 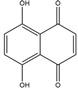 ^	Not reported	iodogen	[^131^I]napthazarine 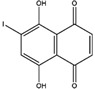	HPLC with retention time of 8.53 min Radiochemical Purity: >95% (by HPLC)	[60]
Plumbagin 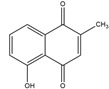	Not reported	iodogen	[^131^I]plumbagin 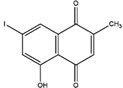	Radiochemical Purity: >95% (by TLRC)	[60]
Juglone 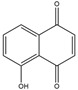	Not reported	iodogen	[^131^I]juglone 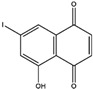	Radiochemical Purity: >95% (by TLRC)	[60]
Resveratrol ^ 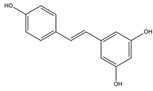 ^	Structure was characterized by NMR LC MS [M+H]+ with *m*/*z* of 229.09	iodogen	[^131^I]resveratrol 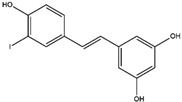	Structure was characterized by NMR Radiochemical Purity: >95% (by TLRC)	[67]
Genistein	Not reported	chloramine-T	[^131^I]genistein Predicted structure have not reported	Not reported Radiochemical Purity: >95% (by TLRC)	[71]
6-Gingerol	Not reported	iodogen	[^131^I]6-gingerol Predicted structure have not reported	Radiochemical Purity: >95% by TLRC)	[74]
6-Shogaol	Not reported	iodogen	[^131^I]6-shogaol Predicted structure have not reported	Radiochemical Purity: >95% (by TLRC)	[74]
Thymoquinone	Characterized by FTIR has C-H (2950–2800 cm^−1^), C=C aromatic (1625–1440 cm^−1^) and C=O ketones (1700–1665 cm^−1^)	iodogen	[^131^I]thymoquinone Predicted structure have not reported	Radiochemical Purity: <95% (by TLRC)	[75]
FATQCSNPs	Characterized by FTIR has amine stretch in Chitosan (3550–3250cm^−1^), OH from Folic acid (3200–2500 cm^−1^), C = O ketones from thymoquinone (1690 cm^−1^), C = O carboylic acid from Folic acid (1715 cm^−1^), C-C (1300–1100 cm^−1^) and C-O (1320–1210 cm^−1^)	iodogen	[^131^I]FATQCSNPs Predicted structure have not reported	Radiochemical Purity < 95% (by TLRC)	[75]
Rutin	Not reported	chloramine-T	[^131^I]rutin	Radiochemical Purity: <95% (by TLRC)	[78]
Ferulic acid ^ 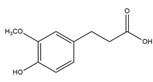 ^	Not reported	chloramine-T	[^131^I]ferulic acid 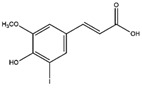	LC/MS showed *m.z* 321.02 HPLC with retention time 17 min Radiochemical Purity: >95%	[82]
Khellin ^ 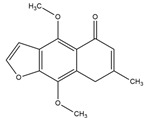 ^	Not reported	iodogen	[^131^I]khellin 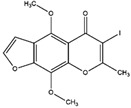	Radiochemical Purity: >95% (by HPLC)	[83]
Zearalenone ^ 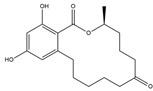 ^	HPLC with retention time of 14.7 min	chloramine-T	[^125^I]I-zearalenone 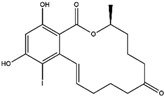	HPLC with retention time of 15.8 min Purity: >95% (by HPLC)	[86]
Riboflavin ^ 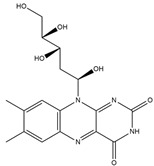 ^	Not reported	iodogen	[^131^I]I-riboflavin 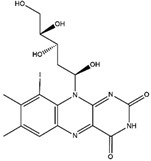	Radiochemical Purity: >95% (by paper chromatography)	[89]
Shikonin ^ 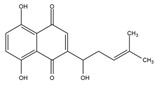 ^	Not reported	chloramine-T	[^131^I]shikonin 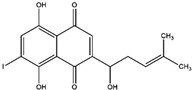	Structure was characterized by NMR HPLC Rt of 8.14 Radiochemical Purity: <95%	[92]

**Table 4:** Evaluation and characterization of natural compound-based radiopharmaceutical candidates against with iodine radioisotopes.

Compound	Stability	Log P	Cell Uptake	Preclinical Study	Dosimetry	Refs.
[^131^I]hydroxytyrosol	<4 h	−0.41 ± 0.12	Cellular uptake on Hutu80 (37.10%) > Caco2 (27.80%) > MCF7 (14.9%) > PC3 (14.50%)	Biodistribution: highest uptake in bladder, stomach, and intestine.	Not reported	[26]
[^125^I]khellin	>24 h	Not reported	Not reported	Biodistribution: The highest uptake in heart, lung, and spleen.	Not reported	[28]
[^131^I]hypericin	Not reported	Not reported	Not reported	Biodistribution: low uptake in necrosis cells but higher in lung, spleen, liver	High absorbed radiation dose in necrotic tissues.	[29,30]
[^123^I]hypericin	Not reported	Not reported	Not reported	Biodistribution: high uptake in necrosis cells but lower in lung, spleen, liver.	High absorbed radiation dose in necrotic tissues.	[30,31]
[^131^I]lawsone	<4 h	−0.26 ± 0.06	Keratinoccyte (25.46%) > BJ (5.43%) > MCF7 (5.32%) > Caco2) (5.28%) on 4 h	Biodistribution: highest uptake in uterus, breast and ovary (female mice); and prostate (male mice)	Not reported	[33]
[^125^I]homoisoflavonoids	Not reported	Not reported	Not reported	Biodistribution in normal mice: high uptake in the brain with rapid clearance from the brain.	Not reported	[34]
[^131^I]GFLAS	>24 h	−0.99 ± 0.03	Cellular uptake on PC3 > MCF7	not reported	Not reported	[36]
[^131^I]sennidin A	In vivo stability > 48 h	−1.11 ± 0.02	Not reported	Pharmacokinetics: AUC of 634.65 MBq/Lxh, clearance 0.02 L/h/kg. The elimination half-life (t_1/2_) of 11.75 hours SPECT/CT image shows high accumulation of radioactivity in necrotic tissue. Biodistribution: high uptake in necrotic tissues, liver, spleen and kidney	Not reported	[37]
[^131^I]protohypericin	Not reported	Not reported	Not reported	Biodistribution: the highest ratio of target/non-target tissues was 11.7 Pharmacokinetics: concentration after injection in blood 99.451±4.442 MBq/L t_1/22_ was 14.9 h using noncompartmental analyses (show fast blood clearance) SPECT-CT, autoradiography, and histological staining showed high uptake in necrotic tissues	Not reported	[38]
[^131^I]sennoside B	Not reported	Not reported	Not reported	SPECT-CT showed selective accumulation of radioactivity in the necrotic tissues. The highest biodistribution: the highest uptake in necrotic liver, necrotic muscle and kidney Pharmacokinetics t_1/2_ 8.6 h (fast clearance from blood)	Not reported	[3]
[^123^I]hesperetin	<4 h	Not reported	Not reported	The highest Biodistribution: highest uptake in stomach and intestine.	Not reported	[42]
[^125^I]rutin	Not reported	Not reported	Not reported	Biodistribution and SPECT/CT studies in mice oral administration: high biodistribution uptake in stomach and small intestine intravena administration: highest biodistribution uptake in liver and small intestine	Not reported	[44]
[^131^I]rhein	>24 h	Not reported	Not reported	Stability > 24 h Pharmacokinetics: t_1/2_ 8.2 ± 0.49 h Biodistribution: has optimum heart-to-blood, heart-to-liver and heart-to-lung ratios.	Not reported	[47]
[^131^I]eugenol	In vivo stability > 48 h	−1.50 ± 0.15	In 4 h, cellular uptake on PC3 (54.35%)> MCF7 (45.68%)> Caco-2 (36.60%)	Not reported	Not reported	[51]
[^131^I]quercetin		Not reported	Cellular uptake in human thyroid: TT cell lines> FTC-133 cell lines> DRO cell lines Cells viability study with CCK-8 assay showed the rate of proliferation inhibiton of [^131^I]I-qQuercetin ≥ [^131^I^+^]qQuercetin > qQuercetin > iodine-131^131^I	Biodistribution: the highest biodistribution uptake in tumors. In vivo therapeutic efficacy study in tumors showed that a single dose can suppressed suppress tumor growth with mild side effects.	Not reported	[54]
[^131^I]arbutin		Not reported	Not reported	The biodistribution study in CT26 tumor model mice were showed the highest uptake in bladder and kidney	Not reported	[57]
[^131^I]vitexin		1.48 ± 0.06	Not reported	Pharmacokinetics: t_1/2_ 5.3 h Biodistribution: necrotic-viable myocardium ratio of 5.0 ± 0.9 SPECT/CT: clear necrosis imaging on CA4P-treated W256 tumors. In vivo blocking study: could be blocked 51.95% and 64.29% by EB and cold vitexin	Not reported	[58]
[^131^I]napthazarin		Not reported	Not reported	Biodistribution: high necrotic-to-viable ratio and necrosis-to-blood ratio Pharmacokinetic: t_1/2_ 4.73 h SPECT/CT: necrotic myocardium could be clearly visualized in vitro DNA-binding: napthazarin could bind to DNA through intercalation in vivo blocking study: necrotic muscle could be significantly blocked by excessive ethidium bromide (a typical DNA intercalator) and cold naphthazarin with 63.49 and 71.96% decline.	Not reported	[60]
[^131^I]plumbagin	>12 h	Not reported	Not reported	Biodistribution: exhibited higher DNA-binding 5.60 × 10^4^ M^−1^	Not reported	[60]
[^131^I]juglone	>12 h	Not reported	Not reported	Biodistribution: exhibited higher DNA-binding: 7.53 × 10^4^ M^−1^	Not reported	[60]
[^131^I]resveratrol	>24 h	0.48 ± 0.2	Cellular uptake on human neuroblastoma cell lines SK-N-AS (24.24%)> SH-SY5Y (15.04%)	Not reported	Not reported	[67]
[^131^I]genistein				Evaluation have not reported		[71]
[^131^I]6-gingerol	Not reported	Not reported	Cellular uptake in breast cancer cell lines MDA-MB-231: [^131^I]-6-sShogaol > [^131^I]-6-gGingerol	Not reported	Not reported	[74]
[^131^I]6-shogaol	Not reported	Not reported	Cellular uptake in breast cancer cell lines MCF7: [^131^I]-6-sShogaol similar to [^131^I]-6-g-Gingerol	Not reported	Not reported	[74]
[^131^I]thymoquinone	4 h	Not reported	Cellular uptake: SKOV3 (7.3%) > Caco-2 (5.75%) (in dose 200–1000 ng/mL)	Not reported	Not reported	[75]
[^131^I]FATQCSNPs	4 h	Not reported	Cellular uptake: SKOV3 (12.38%) > Caco-2 (6.73%) (in dose 200–1000 ng/mL)	Not reported	Not reported	[75]
[^131^I]rutin	Not reported	0.44 ± 0.16	Not reported	Not reported	Not reported	[78]
[^131^I]ferulic acid	>24 h	Not reported	Not reported	Biodistribution: %ID/gram in tumor s 4.35 ± 0.41 with tumor to muscle ratio 2.79	Not reported	[82]
[^131^I]khellin	>24 h	Not reported	Not reported	Biodistribution: the highest uptake in kidney, liver, intestine, tumor	Not reported	[83]
[^125^I]zearalenone	>24 h	Not reported	Not reported	Biodistribution in normal and bearing acid lactic mice showed a high accumulation in blood, liver, kidney, and intestine	Not reported	[86]
[^131^I]riboflavin	Not reported	Not reported	Not reported	SPECT/CT image: uptake in the cerebral injury> normal brain Autoradiography: infarcted to normal brain ratio 3.63 Blocking study: infarcted to normal brain ratio decrease to 1.98 after blocking	Not reported	[89]
[^131^I]shikonin	Not reported	Not reported	Not reported	Biodistribution the highest uptake in lung tissue (81.28% ID/g) Pharmacokinetics: t_1/2_ elimination 40.05 ± 3.02 min.	Not reported	[92]

**Table 5:** Challenges in the development of natural product-based radiopharmaceuticals.

No	Challenges	Cases on Previous Studies
1.	Problem related to radiochemical purity	Radiolabeled compounds have low radiochemical purity (RCP < 95%): [^125^I]khellin, [^131^I]lawsone, [^131^I]GFLAS, [^131^I]sennidin A, [^131^I]thymoquinone, [^131^I]FATQCSNPs, [^131^I]rutin, and [^131^I]shikonin
2.	Problem related to biodistribution	The biodistribution pattern was high in certain organs, especially the thyroid, intestine and stomach: [^131^I]hydroxytyrosol, [^123^I]hesperetin, [^125^I]rutin, [^131^I]khellin, and [^125^I]zearalenone

**Table 6:** Critical points and considerations of radioiodination reaction method as a strategy for increasing the radiochemical purity.

Radioiodination Method	Critical Point that Needs to Be Optimized	Considerations	Refs.
*Electrophilic substitution*			
Chloramine-T (CAT)	pH CAT concentration Temperature	pH should be neutral, weak acid, or weak basic media.	[28,109,110,111,112,113,114,115]
		Excessive concentration causes oxidative side reactions such as polymerization, chlorination, and denaturation of the substrate.	
		Temperature to achieve the energy required for substitute H^+^ from the aromatic ring with radioactive iodonium ion.	
Iodogen	pH Iodogen concentration Solvent	pH should be 7–8	[116,117,118,119]
		excessive concentration causes precipitates on the walls of the reaction vessel causing a low radiochemical purity.	
		Solvent: substrate in DMSO solvent showed with higher radiochemical purity RCP than substrate in aqueous solvent.	
N-halosuccinimides (N-chlorosuccinimide and N-iodosuccinimide)	pH Mediators		
		pH: N-iodosuccinimide with high activity in a strong acid medium	[93,120,121]
		Mediators such as NGA or mAB	
*Nucleophilic Substitutions* (halogen and isotopic exchange)	Temperature Reaction time	High temperature is required	[93]
		Reaction time: reactions take a long reaction time	

**Table 7:** The resistance of some radioiodinated groups against in vivo deiodination [129].

Resistant to Deiodination	Non-Resistant to Deiodination
Iodinated carbon sp^2^	Iodinated carbon sp and sp3
Iodoarenes	Iodoaniline
Iodovinyl	Iodophenols
Iodoallyl	Radioiodinated nitrogen-containing (quinozalines, indoles, or imidazoles), and sulfur-containing (thiophenes) heterocycles
Radioiodinated oxygen-containing heterocycles	

## Data Availability

Not applicable.
